# Real Life Use of Bendamustine in Elderly Patients with Lymphoid Neoplasia

**DOI:** 10.3390/jpm11040249

**Published:** 2021-03-30

**Authors:** Irene Dogliotti, Simone Ragaini, Francesco Vassallo, Elia Boccellato, Gabriele De Luca, Francesca Perutelli, Carola Boccomini, Michele Clerico, Barbara Botto, Daniele Grimaldi, Lorella Orsucci, Simone Ferrero, Candida Vitale, Dario Ferrero, Marta Coscia, Federica Cavallo

**Affiliations:** 1 Stem Cell Transplant Unit, A.O.U., Città della Salute e della Scienza di Torino, 10126 Turin, Italy; irenedogl@hotmail.com; 2 Division of Hematology, Department of Molecular Biotechnology and Health Sciences, University of Torino, A.O.U., Città della Salute e della Scienza di Torino, 10126 Turin, Italy; simone.ragaini@unito.it (S.R.); elia.boccellato@gmail.com (E.B.); gabriele.deluca@unito.it (G.D.L.); francescaperutelli@gmail.com (F.P.); michele.clerico@gmail.com (M.C.); dgrimaldi@cittadellasalute.to.it (D.G.); simone.ferrero@unito.it (S.F.); candida.vitale@unito.it (C.V.); dario.ferrero@unito.it (D.F.); marta.coscia@unito.it (M.C.); 3 Division of Hematology, A.O.U., Città della Salute e della Scienza di Torino, 10126 Turin, Italy; fvassallo@cittadellasalute.to.it (F.V.); cboccomini@cittadellasalute.to.it (C.B.); bbotto@cittadellasalute.to.it (B.B.); lorsucci@cittadellasalute.to.it (L.O.)

**Keywords:** bendamustine, lymphoma, chronic lymphocytic leukemia, elderly, safety, efficacy

## Abstract

Background. Bendamustine is a cytotoxic alkylating drug with a broad range of indications as a single agent or in combination therapy in lymphoid neoplasia patients. However, its tolerability in elderly patients is still debated. Methods: An observational, retrospective study was carried out; patients with chronic lymphocytic leukemia (CLL) or lymphoma, aged ≥ 65 years old, treated with bendamustine-based regimens in first or subsequent lines between 2010 and 2020 were considered eligible. Results: Overall, 179 patients aged ≥ 65 years were enrolled, 53% between 71 and 79 years old. Cumulative Illness Rating Scale (CIRS) comorbidity score was ≥6 in 54% patients. Overall survival (OS) at 12 months was 95% (95% confidence interval [CI]: 90–97%); after a median follow up of 50 months, median OS was 84 months. The overall response rate was 87%, with 56% complete responses; the median time to progression (TTP) was 61 months. The baseline factors affecting OS by multivariable analysis were sex, histological diagnosis, renal function, and planned bendamustine dose, while only type of lymphoma and bendamustine dose impacted on TTP. Main adverse events were neutropenia (grade ≥ 3: 43%) and infections (any grade: 36%), with 17% of patients requiring hospital admission. Conclusions: The responses to bendamustine, as well as survival, are relevant even in advanced age patients, with a manageable incidence of acute toxicity.

## 1. Background

Bendamustine is a uniquely structured cytotoxic alkylating drug, that has been broadly used in the field of B-cell lymphoprolipherative neoplasia.

Bendamustine is administered intravenously, and is mainly catabolized via non-enzymatic hydrolysis (although a small fraction is also a substrate for cytochrome P450, isoform 1A2) into active circulating metabolites [1]. Interestingly, thanks to its short half-life and metabolic pathways, renal or hepatic function seem to have a limited impact on bendamustine exposure, and no particular dose reduction is needed for mild to moderate impairments.

Extensive clinical experience with bendamustine is now available; in indolent non Hodgkin Lymphoma (iNHL) and mantle cell lymphoma (MCL) it is currently employed in association with anti CD20 monoclonal antibodies, both in first and second line, while in Hodgkin Lymphoma (HL) it is used as single agent or in combination with polychemotherapy in relapsed or chemotherapy-refractory patients [2].

Nowadays, new targeted drugs have extensively replaced bendamustine-containing regimens for the treatment of patients with chronic lymphocytic leukemia (CLL). However, the combination bendamustine plus rituximab (BR) can still be employed for the frontline treatment of patients ineligible to fludarabine containing regimens and with a low risk disease (i.e., patients without TP53 abnormalities and with mutated immunoglobulin variable heavy chain gene (IGHV) [3]. In relapsed patients with CLL, the combination venetoclax plus rituximab was superior to BR, both in terms of Progression Free Survival (PFS) and Overall Survival (OS) [4].

In the frontline setting, a recent phase III randomized study comparing BR with the Bruton tyrosine kinase inhibitor (BTKi) ibrutinib, has shown a significant benefit for ibrutinib-treated patients in terms of PFS and safety outcomes [5], but not in terms of OS. Similarly, a matched-adjusted indirect comparison of patients who had received BR or ibrutinib as first salvage treatment outside of clinical trials showed no OS difference [6]. More recently, a retrospective study has demonstrated that BR is an effective first-line regimen in unfit patients (largely consisting of elderly patients aged > 65 years), with the exception of patients with unfavorable genetic features (i.e., del(17p)/TP53 aberrations and IGHV-unmutated status) [7]. However, the safety and efficacy of bendamustine-containing regimens according to age groups of patients with CLL is so far unexplored. 

BR demonstrated high efficacy in non Hodgkin Lymphoma (NHL) in two phase III trials; the Study group indolent Lymphomas (StiL) study compared BR with rituximab-cyclophosphamide, doxorubicin, vincristine, and prednisone (R-CHOP) in iNHL and MCL, showing longer PFS (69 vs. 31 months, *p* < 0.0001) and improved complete response (CR) rates for BR, with no difference in OS and comparable incidence of secondary malignancies [8,9].

The BRIGHT study (study of Bendamustine Hydrochloride and Rituximab compared with R-CVP or R-CHOP in the first-line treatment of patients with advanced iNHL or MCL), in a similar patient population, showed non-inferiority of BR to R-CHOP in terms of CR rates (31 vs. 25%, *p* = 0.0225), with a long-term benefit of BR on PFS (65 vs. 55% at 5 years), and highlighted the different safety profiles of the two regimens [10,11].

Bendamustine is usually regarded as a tolerable regimen, less toxic compared to other classical chemotherapy combinations; evidence from clinical trials report that the most common grade 3–4 toxicity events related to bendamustine are nausea, vomiting, skin rash, fatigue, myelosuppression and infections [7,8,9,12]. Recent systematic reviews showed that incidence of such events with bendamustine was not significantly superior than with R-CHOP, chlorambucil or rituximab-fludarabine, and cyclophosphamide (R-FC) regimens [13,14]; however, special interest has been raised on the incidence of infections in bendamustine-exposed patients, particularly for atypical agents like cytomegalovirus (CMV) and Pneumocystis Jirovecii (PJ) [15,16,17,18]. 

Moreover, the impact of bendamustine dose on adverse events, in particular for elderly patients, still needs to be determined, with initial reports suggesting that a lower dose (from 45 to 60 mg/m^2^) might be beneficial in reducing the prevalence of infections and nausea. 

## 2. Aims

This study aims at dissecting the real-life applicability of bendamustine in a broad setting of lymphoid neoplasms, including iNHL, MCL, and HL. We focused on determining the balance between efficacy and safety of bendamustine-based regimens according to increasing age ranges.

## 3. Materials and Methods

An observational, single center retrospective study was carried out; all consecutive patients diagnosed between 2010 and 2020 with CLL or lymphoma, aged ≥ 65 years old and treated with bendamustine in first or subsequent lines, either as single agent or in combination with anti CD20 antibodies or other agents, were eligible for the study. 

In order to better reflect the complexity of the bendamustine-exposed population, no exclusion criteria were applied, other than age < 65 years old at the time of bendamustine administration.

Bendamustine-based treatment was allocated to all patients per local clinical practice; in particular, a tailored treatment schedule was selected based on patients’ age, comorbidity, fitness, and available histology and molecular biology data, as well as on current indications in Italy.

Bendamustine was administered at 90, 70, or <70 mg/m^2^, depending on clinicians’ judgement, on days 1, 2, of 28- day cycles; co-administration with anti CD20 antibodies or other chemotherapy agents was decided after multidisciplinary case discussion. The primary endpoint of the study was OS at 12 months from initiation of bendamustine-based treatment considering both the expected advanced age of enrolled patients and the diagnosis variabilityof the potential cohort of patients that included both indolent and aggressive diseases. Secondary endpoints included time to progression (TTP) and response rates, incidence of treatment-related adverse events (AE); both acute and late toxicity was evaluated, including hematological and non-hematological events, and secondary malignancies.

Disease staging at bendamustine initiation was performed according to Ann Arbor staging system [19] and Rai staging [3] for patients with lymphoma and CLL, respectively.

AEs were categorized and graded according to the National Cancer Institute Common Terminology Criteria for Adverse Events (version 5.0), except for hematological toxicities of patients with CLL, which were graded in accordance with the International Workshop on Chronic Lymphocytic Leukemia (*iwCLL*) iwCLL grading scale [20]. 

TTP was measured from the date of bendamustine treatment initiation to the date of progression, relapse, or death. OS was measured from the date of bendamustine treatment initiation to the date of death from any cause. Response assessment technique and criteria were defined as per clinical practice according to specific histology and guidelines at the time of bendamustine treatment; the response of CLL to treatment was defined based on the international workshop on CLL (iwCLL) guidelines [20].

All alive patients signed informed consent for the study, that was conducted according to Helsinki’s declaration [21]. Approval of this study was obtained from the local Ethic Committee of the University Hospital of Torino, Italy (identification n. 00382/2020).

Electronic health records of the two Divisions of Hematology of the promoting center were analyzed by study personnel and data about patients’ demographics, disease characteristics, significant comorbidities, treatments received, adverse events and outcome were collected.

Statistical analysis was carried out using STATA 13 to estimate survival with the Kaplan Meier method and the log rank test was applied to compare the survival distributions of the samples. For the other analyses, R v 4.0.0. tool was used.

The Kruskal–Wallis test for continuous variables was used to compare medians between groups; for categorical variables, the chi-squared test or Fisher’s exact test was used.

The Cox proportional hazards model was implemented for the univariate and multivariate survival analysis. The variables for the multivariate were selected based on significance at univariate model and on clinical criteria.

## 4. Results

### 4.1. Patients and Treatments

Overall, 179 patients aged ≥ 65 years old received bendamustine-based treatment for lymphoprolipherative diseases within the time window considered (2010–2020). 

Details on patients’ distribution and characteristics are resumed in Table 1 and in Supplementary Tables S1 and S2.

Briefly, 51 (28.5%) were diagnosed with CLL, while 43 (24%) had follicular lymphoma (FL), 41 (23%) MCL, 22 (12%) marginal zone lymphoma (MZL), 16 (9%) lymphoplasmacytic lymphoma/Waldenström macroglobulinemia (LPL/WM); in total, four patients were treated for aggressive lymphoma (1 diffuse large B-cell lymphoma [DLBCL], 3 HL), 2 had other histotypes of indolent lymphoma.

Median age at bendamustine administration was 74 years, with 53% of patients in the range of 71 to 79 years old. A total of 81 patients (45%) were female, 96 patients (54%) had a CIRS score ≥ 6, and 16 (9%) had at least 1 CIRS item ≥ 4, indicating a severe comorbidity. Creatinine clearance (estimated by Cockroft–Gault equation) before therapy initiation was below 50 mL/min in only 14% of patients. Overall, 12 out of 30 evaluable patients had documented p53 disruption at the time of bendamustine administration.

As patients were not selected according to line of therapy, 131 (73%) patients were treated in first line, 32 (18%) in second line, and 16 (9%) in more advanced lines; a total of 3 patients had received prior autologous stem cell transplantation.

Bendamustine was administered in combination therapy with anti CD20 antibodies (rituximab, *n* = 160, or obinutuzumab, *n* = 2) in 90% of patients, while 17 patients did not receive anti CD20; polychemotherapic regimens (namely, bendamustine-cytarabine (BAC), bendamustine-gemcitabine-vinorelbine (BEGEV), as well as other schemes within clinical trials) were delivered in 33 (18%) patients. Initially planned bendamustine dose was 90 mg/m^2^ in 106 (61%) patients, 70 mg/m^2^ in 58 (33.5%), and below 70 mg/m^2^ in only 9 patients. A total of 150 patients completed at least 4 courses of the planned regimens, while 120 reached 6 cycles completion. Actually, 37 (21%) of elderly patients undergoing bendamustine-containing regimens had to discontinue treatment due to toxicity or progressive disease, while 65 (37%) had a treatment delay of at least 1 week between cycles, and, for 23 (13%), a dose reduction was required due to toxicity events (Table 2); interestingly, treatment discontinuation affected mostly CLL patients, whereas treatment delay and dose reduction differences between different histology groups were minor and not statistically significant (as shown in Supplementary Table S2). 

In this real-life study, prophylactic medications for cytopenia and infections were not pre-defined, but administered concomitantly to bendamustine regimens according to local practice and clinicians’ decision. Granulocyte colony-stimulating factor (G-CSF) as primary prophylaxis was employed in 77 (43%) patients, human recombinant erythropoietin (rhEPO) was used due to disease-related anemia in 29 (16%) patients.

Trimethoprim-sulfamethoxazole prophylaxis for Pneumocystis jirovecii lung infection was given in 146 out of 179 patients, while acyclovir prophylaxis for varicella zoster virus reactivation in 72 (41%).

Among patients requiring further lines of therapy for their lymphoprolipherative disease, 36% received intravenous chemotherapy regimens, 30% proceeded to targeted drugs (either BTKi or phosphoinositide 3-kinase inhibitors (PI3Ki)), around 8% received radiation therapy, nearly 8% anti CD20 as single agent, and 17% received palliative oral chemotherapy.

### 4.2. Efficacy Outcomes

#### 4.2.1. Overall Response Rate

**Figure 1:**
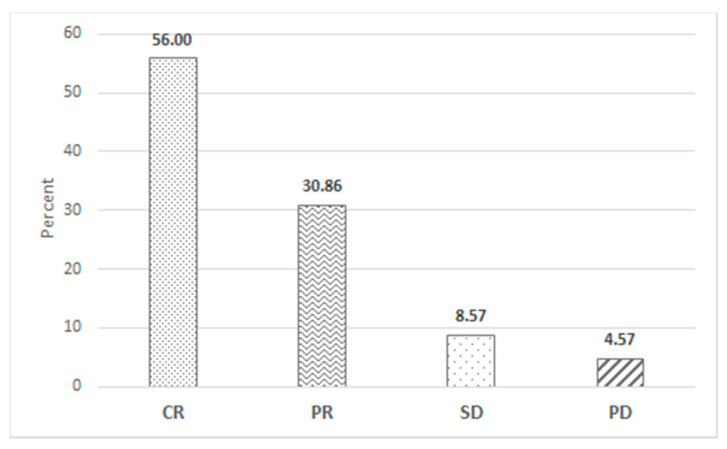
Response to bendamustine treatment. Best response obtained after receiving at least 1 bendamustine-containing course.

Objective response after bendamustine-based treatments was evaluated with positron emission tomography (PET) in 64 (36%) lymphoma patients, and with computed tomography (CT) scan in 75 (43%), chest X-ray and abdomen ultrasound in 18 (10%), and by clinical evaluation only in 19 (11%) CLL and lymphoma patients; best response obtained, irrespective of histological diagnosis or treatment line, was CR in 56%, partial response (PR) in 31%, stable disease (SD) in 9%, and progressive disease (PD) in 5% (Figure 1).

#### 4.2.2. Overall Survival 

**Figure 2:**
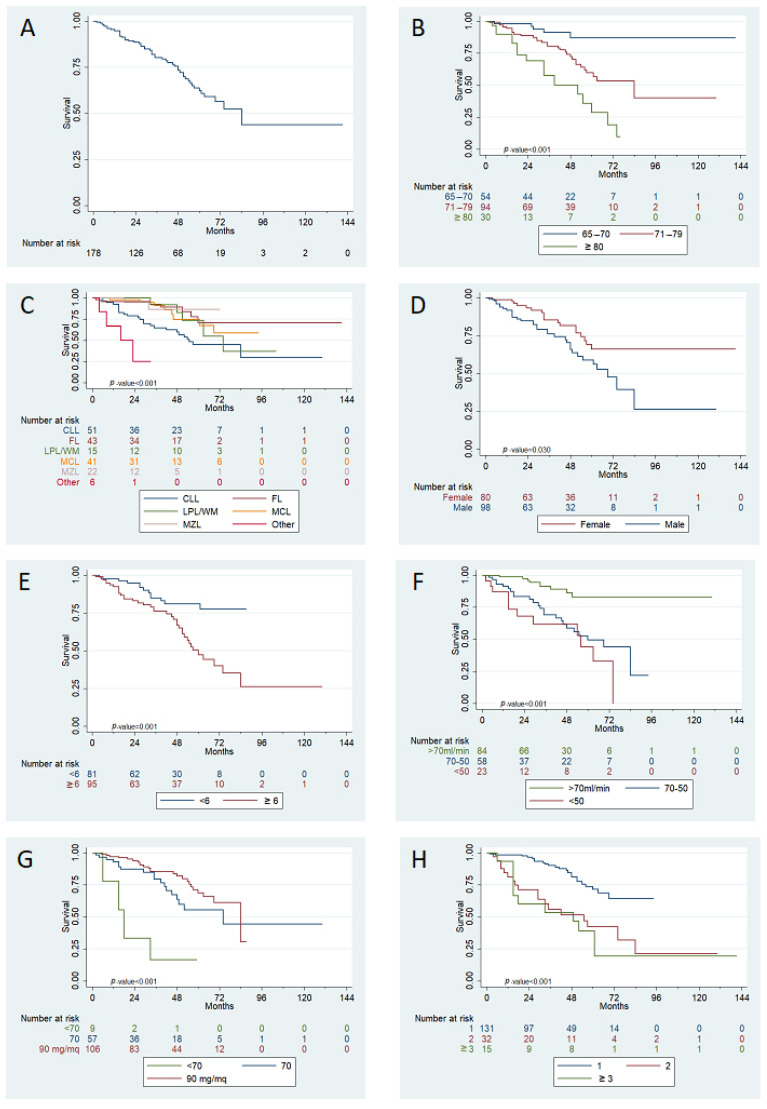
Univariate analysis for OS. panel (**A**): median OS after bendamustine-based treatment initiation, overall series; panel (**B**): OS according to different age classes; panel (**C**): OS stratified accoding to initial diagnosis; panel (**D**): OS divided by patients’ sex; panel (**E**): OS according to CIRS score; panel (**F**): according to BCC at baseline; panel (**G**): effect of bendamustine dose received on OS; panel (**H**): OS stratified according to line of therapy at bendamustine treatment.

After a median follow up of 50 months, the estimated median OS of the overall series was 84 months; OS at 12 months (primary endpoint of the study) was 95% (95% confidence interval, 95%CI, 90–97%), with OS at 4 years of 73% (95%CI 65–80%, Figure 2). 

When stratified according to age distribution, median OS was not reached for patients 65–70 years old at the time of bendamustine initiation; it was 84 months for patients 71–79 years old, and 52 months for patients ≥80 years old (*p* < 0.001). Median OS was not reached for female patients, while for male patients it was 69 months (OS at 4 years: 82 vs. 66%, *p* = 0.030). 

OS at 4 years was: 89% for FL patients, vs. 86% for MZL, 82% for LPL/WM, 74% for MCL, and 59% for CLL patients (*p* < 0.001).

According to baseline comorbidities, patients with a CIRS score of 6 or higher presented inferior OS compared to those with a lower comorbidity burden (80 vs. 67% at 4 years, *p* = 0.001); however, there was no statistical difference in OS for patients with at least one comorbidity considered as serious, compared to patients with only mild to moderate comorbidities. There was also no difference in baseline comorbidities between different histology groups. Interestingly, a preserved renal function seemed to be statistically correlated with OS (86% at 4 years for patients having a baseline creatinine clearance (BCC) > 70 mL/min, 59% for BCC between 70–50 mL/min and 62% for BCC < 50 mL/min (*p* < 0.001). 

Planned bendamustine dose was also significantly associated with OS: 4y-OS was 82 vs. 63 vs. 17% for 90, 70, and <70 mg/m^2^, respectively (*p* < 0.001); in terms of lines of treatment, administration of bendamustine as first line was clearly superior to second and subsequent lines (median OS not reached vs. 55 vs. 49 months). 

Of note, adoption of G-CSF and trimethoprim-sulfamethoxazole prophylaxis both impacted favorably on OS, and presence of hematologic toxicity (any grade) was associated with poorer OS (Supplementary Figure S1). 

Dose delays >1 week or unplanned bendamustine dose reductions as well as occurrence of admissions for any reason or infectious events of any grade did not affect patients’ OS, regardless of their histological diagnoses (*p* = 0.794, 0.580, 0.093, 0.694, respectively, data not shown). However, early treatment interruption due to toxicity or progressive disease negatively impacted on OS (*p* < 0.001, Supplementary Figure S1), as well as diagnosis of a second neoplasm after treatment with bendamustine (*p* = 0.032) (Supplementary Figure S1). 

**Figure 3:**
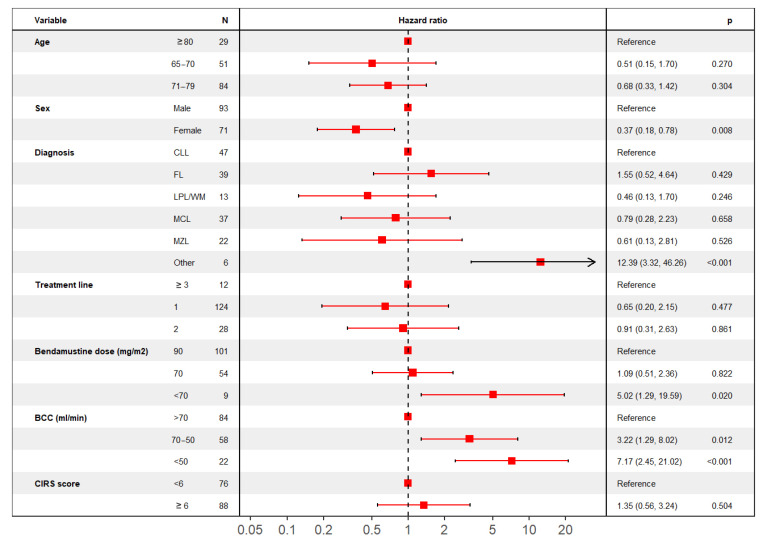
Multivariable analysis for OS. Forest plot describing multivariable analysis results for OS.

In a multivariable model considering only variables that were deemed significant for univariate analysis, a significant impact on OS was confirmed for patients’ sex, diagnosis, BCC before treatment initiation, and planned bendamustine dose (Figure 3), while age (stratified into three groups), line of treatment and CIRS comorbidity score were not shown to affect OS. 

#### 4.2.3. Time to Progression

**Figure 4:**
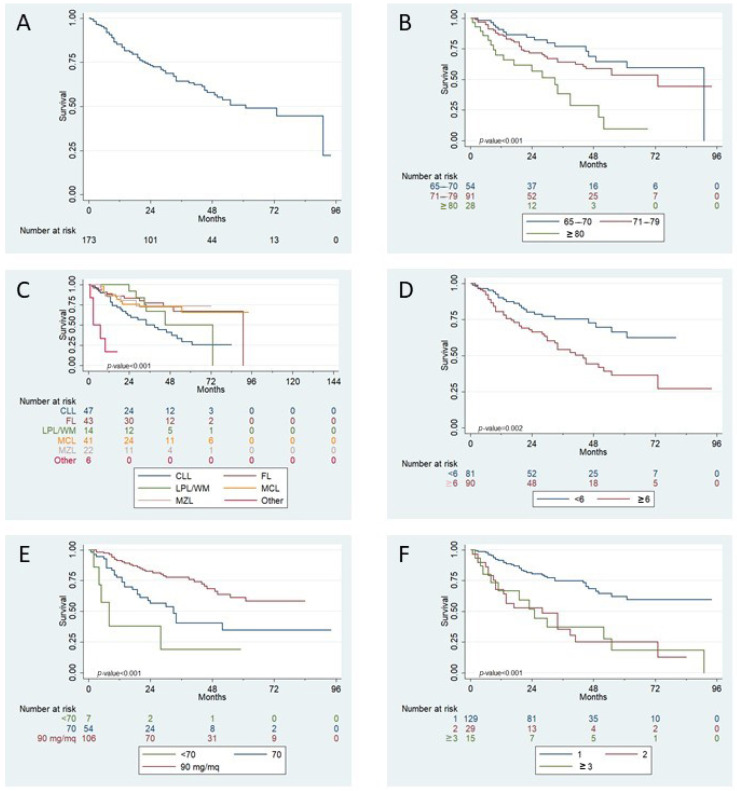
Univariate analysis for TTP. Panel (**A**): median TTP after bendamustine-based treatment initiation, overall series; panel (**B**): TTP according to different age classes; panel (**C**): TTP stratified by initial histological diagnosis; panel (**D**): TTP according to CIRS score at bendamustine administration; panel (**E**): effect of bendamustine dose received on TTP; panel (**F**): TTP stratified according to line of therapy at bendamustine treatment.

After a median follow up of 45 months, median TTP for the overall cohort was 61 months (Figure 4).

TTP at 12 months was 85% (95%CI 79–90%), while at 4 years TTP was 58% (95%CI 49–66%).

At four-years, TTP, according to age class, was 69 vs. 59 vs. 28% in patients aged 65–70, 71–79, and ≥80 years old, respectively (*p* < 0.001). At four-years, TTP was 73% for FL and MZL patients, 72% for MCL, 50% for LPL/WM, and 41% for CLL patients (*p* < 0.001). Patients with a CIRS score ≥ 6 had a median TTP of 44 months vs. not reached for patients with lower comorbidity index (*p* = 0.002). 

Similar to OS, better TTP was significantly related to with bendamustine dose (4y- TTP was 68 vs. 40 vs. 19% for 90, 70, and below 70 mg/m^2^, respectively, *p* < 0.001), as well as for line of treatment (median TTP: not reached vs. 28 vs. 25 months, *p* < 0.001) and need for therapy interruption for any cause (median TTP: 91 vs. 19 months, *p* < 0.001, Supplementary Figure S2). 

**Figure 5:**
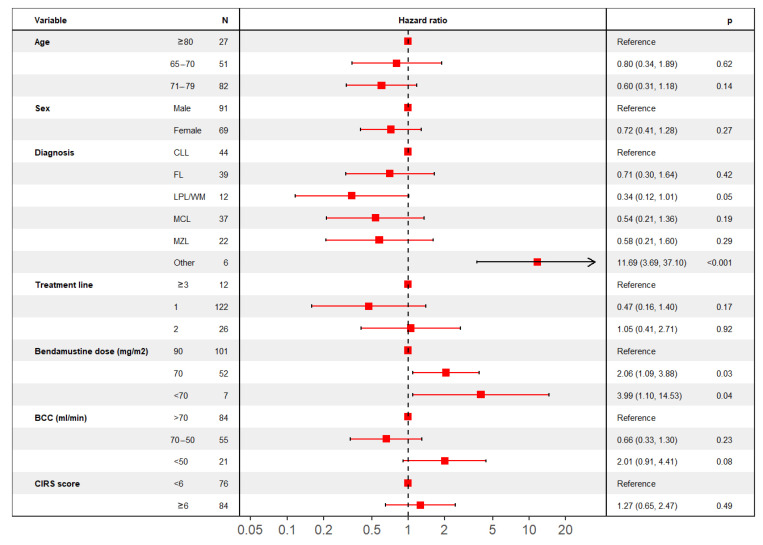
Multivariable analysis for TTP. Forest plot describing multivariable analysis results for TTP.

In multivariable analysis, a significant impact on TTP was shown only for histological diagnosis and planned bendamustine dose (Figure 5). 

### 4.3. Safety

Overall, the regimen was, in general, well tolerated in terms of both hematological and non-hematological toxicity (Table 3) and there was no difference in adverse event incidence among histology subgroups. The most frequent hematological adverse event of any grade was anemia, which occurred in 105 patients (59%). The most frequent hematological grade ≥ 3 toxicity was neutropenia, which occurred in 79 (43%) patients.

As for non-hematological toxicity, 64 patients (36%) had at least one infection event (any grade). Respiratory infections and fever of unknown origin (FUO) were the most frequent among all grades infections involving, 25 (14%) and 13 (7%) patients, respectively.

Conversely, the use of bendamustine as first therapy line (first therapy line vs. third line or more OR = 0.28, *p* = 0.022), baseline hemoglobin level > 10 g/dL (Hb > 10 g/dL vs. ≤10 g/dL OR = 0.41, *p* = 0.029), initial platelets count > 100,000/µL (plts > 100,000/µL vs. plts ≤ 100,000/µL OR = 0.37, *p* = 0.029), and trimethoprim-sulfamethoxazole prophylaxis (prophylaxis vs. no prophylaxis: OR = 0.43, *p* = 0.036) correlated with lower occurrence of infections.

Adverse events requiring hospitalization occurred in 31 (17%) patients. Median time to hospital discharge was 10 days. The most frequent causes of hospitalization were sepsis, occurring in 9 cases (5%), and FUO, involving 5 (2.8%) patients.

In univariate analysis, Eastern Cooperative Oncology Group (ECOG) performance score 1–2 (ECOG 1–2 vs. ECOG 0: OR = 3.32, *p* = 0.021), initial bendamustine dose of 70 mg/m^2^ (70 vs. 90 mg/m^2^: OR = 2.29, *p* = 0.046), dose reduction in the planned dose (reduction vs. no reduction: OR = 3.89, *p* = 0.005) and treatment discontinuation (discontinuation vs. no discontinuation: OR = 5.37, *p* < 0.001) correlated to higher risk of hospitalization.

Overall, 40 (22.3%) patients treated with bendamustine developed second cancers. More precisely, 10 patients (6%, 25% of all cancers) presented a second hematologic cancer, 8 patients (4%) developed urogenital cancer, including prostate, and 8 patients received a gastrointestinal cancer diagnosis. In univariate analysis, second cancer occurrence was associated with higher CIRS score (CIRS score ≥ 4 vs. CIRS score < 4: OR = 4.48, *p* = 0.006), decreased kidney function (BCC 70–50 mL/min vs. BCC > 70 mL/min: OR 5.07, *p* < 0.001) and therapy interruption (interruption vs. no interruption: OR = 2.54, *p* = 0.023). On the other hand, patients who received bendamustine as first therapeutic approach after diagnosis showed lower risk of second cancer development compared to those receiving bendamustine as third or further therapy line (first therapy line vs. ≥third line or more: OR = 0.23, *p* = 0.009). Moreover, focusing on histologic diagnosis, all types of NHL (FL, OR = 0.04, *p* < 0.001; LPL/WM, OR = 0.06, *p* = 0.008; MCL, OR = 0.12, *p* < 0.001; MZL, OR = 0.14, *p* = 0.004) were associated to lower second cancer rates than CLL patients.

## 5. Discussion

The present study was conducted as a retrospective real life registry of elderly (≥65 years old) patients with B-cell lymphoprolipherative diseases treated with bendamustine as single agent or in combination; feasibility, efficacy, and safety were analyzed in order to gather information on outcome of unselected patients treated with bendamustine-based regimens. 

Interestingly, our study shows that in clinical practice, bendamustine was widely employed for a variety of different B cell-neoplasms, as per current treatment indications, and without age limitations (17% of enrolled patients were ≥80 years old at bendamustine start), as far as a curative intent treatment was proposed.

Actually, in this series no pre-specified fitness evaluation was available (Comprehensive Geriatric Assessment [CGA] was not mandatory before bendamustine initiation); thus, data reflecting patients’ fitness could only possibly be inferred by combining baseline comorbidity evaluation, laboratory analysis (cytopenias, creatinine clearance alteration), performance status and age. Of note, all enrolled patients were considered by treating physicians as adequately fit for intravenous (iv) chemotherapy regimens, so a proportion of elderly patients, candidate to palliative care or oral chemotherapy was excluded from the study. Interestingly, however, a subsequent line of therapies after bendamustine included, again, intravenous (IV) chemotherapy regimens in 36% of cases, and 30% received targeted drugs, reflecting the generally persisting fitness of patients even after bendamustine failure or progressive disease. Our study was primarily focused on determining OS outcome after bendamustine-based regimens for elderly patients: with a median observation time of 50 months, median OS of the overall series was 84 months, significantly affected by sex, lymphoprolipherative disease histotype, BCC before treatment initiation, and planned bendamustine dose. The impact on multivariate analysis of sex, due to the known survival advantage in favor of female subjects in the general population, and of disease type and bendamustine dose received, mainly due to a differential effect on responses to treatment, was somewhat expected. Conversely, it is interesting to note the less predictable role of BCC, since bendamustine primary route of elimination is metabolism, not renal elimination. Of note, renal function was here estimated by the Cockroft–Gault equation [22] (reflecting serum creatinine levels, sex, age, and weight) as most frequently employed in our local practice, although different options could have been proposed (namely, CKD-EPI [23] equation is known to better reflect renal function in advanced age patients).

Efficacy analysis in our study showed a median TTP of 61 months, and an overall response rate (ORR) of 87% for the whole cohort; in terms of histotypes, patients with indolent lymphoma or MCL had superior TTP as compared to CLL patients, with a trend towards improved outcome for LPL/WM at multivariate analysis. The only factor, other than diagnosis, that confirmed to impact on TTP was bendamustine dose (90 vs. 70 vs. <70 mg/m^2^).

In the registrative, non-inferiority trial of bendamustine-rituximab (BR) compared to R-CHOP) in iNHL and MCL, 60 patients >70 years old were enrolled [8], and no difference in PFS advantage compared to CHOP was seen according to age groups. Previous experience from the Fondazione Italiana Linfomi (FIL) collaborative group has shown promising response rates with bendamustine, mitoxantrone, and rituximab (R-BM) scheme in elderly (aged 65–80 years) FL patients, with 3-years PFS and OS of 67 and 92%, respectively; however, only patients considered as “fit” after comprehensive geriatric assessment (CGA) were eligible to the study [24].

In CLL, trials with novel agents like BTKi, PI3Ki, and BCL2i were designed to have BR as chemoimmunotherapy-based comparator arm [5,25,26,27]. Results from these studies yielded median PFS values of about 40–43 months in patients receiving BR as frontline treatment [5,12], but only 11 months (4-year PFS: 4.6%) in subsequent lines of therapy [4,28]. Real-life multicentric experiences observed similar, if not slightly better results (median PFS: 45 months in previously untreated CLL, and 25 months in relapsed CLL) [6,7,29] and reflected a good efficacy even for advanced-age patients, showing no difference in clinical responses between 60 and 80 years of age [30].

In terms of hematological toxicity, our study showed 141 (78.8%) hematological toxicity of any grade, and 43% severe neutropenia; supportive care with G-CSF prophylaxis was employed in 43% patients. 

Previous studies have also highlighted that the events most frequently reported were G3–4 neutropenias: the StiL trial registered 37% incidence of neutropenia, but only 3% of anemia and 5% of thrombocytopenia [8]; even higher neutropenia rates were observed in a similar population in the BRIGHT study (49% grade 3–4), despite 29% of patients receiving G-CSF prophylaxis at any cycle. Similar results were described also for CLL patients (G3–4 neutropenia: 40%) [29,31] 

Hospital admission rates during bendamustine treatment in our study was 17%, comparable to literature [24,30]. In univariate analysis, admissions correlated with diagnosis type, bendamustine dose and ECOG PS, while age and CIRS comorbidity index did not affect hospitalization requirements, again, emphasizing the lack of a determining role of age distribution per se in this context.

Second cancers in our study were diagnosed in 21% of patients, a higher incidence than what previously reported in bendamustine-based clinical trials [5]; however, a longer follow up, a more advanced age of our series, as well as number of comorbidities compared to the literature and relative frequency of CLL diagnosis could explain this observation. In fact, in indolent lymphomas treated with bendamustine, patients followed for a minimum of 5 years reached 16–20% of second tumors, possibly related to a prolonged reduction in T cell counts [32,33].

Bendamustine dose adjustments according to patients’ age and comorbidities are quite debated, since there is no uniformly accepted indication on how a reduced dose could still impact on clinical benefit with acceptable toxicity profile. In our study, doses below 70 mg/m^2^ confirmed to negatively impact on PFS and OS, while little survival difference was observed in reducing dose from 90 to 70 mg/m^2^; no difference in infectious events, hematologic, cutaneous, or autoimmune toxicity was noted between 70 and 90 mg/m^2^, while necessity of an unplanned dose reduction negatively impacted on all outcomes.

Importantly, due to the retrospective nature of the analysis, no data on patients’ quality of life (QoL) were available; however, it can be assumed that interpretation of toxicity events, especially regarding hospital admissions, infections or high transfusion burden, could at least partially reflect the actual QoL evaluation. Thus, careful preemptive measures and supportive care, like rhEPO and G-CSF for cytopenia, acyclovir, and trimethoprim-sulfamethoxazole prophylaxis, prompt evaluation of potential signs or symptoms of infection, as well as use of concomitant medications to reduce gastrointestinal adverse events (nausea, constipation), are considered crucial to improve patients’ tolerance and satisfaction to bendamustine treatment.

### Limitations of the Study

Our study presents, however, some limitations: firstly, its retrospective nature and limited follow-up period do not allow for long-term evaluation of OS, especially in indolent lymphoprolipherative diseases (such as CLL and FL) where longer follow-up periods are generally needed to assess treatment impact on OS.

Moreover, this retrospective study needs to be considered in the light of the heterogeneity of the enrolled cohort, comprising patients with different histological diagnosis and at different lines of therapy. For this reason, we included in our study all consecutive patients who received bendamustine at age ≥ 65 years during the enrolment time window, aiming at reducing any selection bias.

The lack of pre-specified fitness and comorbidity criteria for bendamustine indication may also give rise to doubts when selecting patients who could benefit from this treatment the most, even though this shows-however-a good manageability in real-life settings.

Lastly, limited follow-up period did not allow to evaluate some aspects of hematological toxicity, such as prolonged cytopenias, which are sometimes associated with bendamustine (and rituximab) use in elderly patients, even in absence of a myelodysplastic marrow [34].

## 6. Conclusions

Bendamustine-based regimens have a range of hematologic indications and are widely employed in routine clinical practice, even after the introduction of targeted therapies; our real-life study showed that responses, as well as survival, are relevant even in advanced age patients, with a relatively low incidence of acute toxicity, compared with either alkylating agents or fludarabine.

As supported by more and more clinical evidence, age alone should not be considered a determinant factor to allocate curative intent chemotherapy treatments, but it should be evaluated in the context of other features like comorbidity, organ function, performance status, presence of care givers and social connections, and fitness scores. Improved patients selection and supportive care are key to allow even very elderly patients with B-cell neoplasms to be efficaciously and safely treated with bendamustine-based regimens.

## Figures and Tables

**Table 1:** Patients Characteristics. Characteristics of the patients at the time of bendamustine therapy initiation.

Characteristic (*n*. of Analyzed Patients) *	Median (IQR) or *n*. (%)
All patients	179
**Age (median [IQR])**	74.00 (70.00, 78.00)
**Age distribution (%)**	
65–70	54 (30.2)
71–79	94 (52.5)
≥80	31 (17.3)
**Sex (%)**	
Male	98 (54.7)
Female	81 (45.3)
**Diagnosis (%)**	
FL	43 (24.0)
CLL	51 (28.5)
MCL	41 (22.9)
MZL	22 (12.3)
LPL/WM	16 (8.9)
HD	3 (1.7)
DLBCL	1 (0.6)
other	2 (1.1)
**Stage (%) (151)**	
0	12 (7.9)
1	13 (8.6)
2	12 (7.9)
3	17 (11.3)
4	97 (64.2)
**Symptoms (%) (158)**	
A	130 (82.3)
B	28 (17.7)
**Total lines (%) (178)**	
1	97 (54.5)
2	31 (17.4)
≥3	50 (28.1)
**Previous ASCT (%) (178)**	
No	175 (98.3)
Yes	3 (1.7)
**Bendamustine treatment line (%)**	
1	131 (73.2)
2	32 (17.9)
≥3	16 (8.9)
**BCC at bendamustine treatment start (%) (166)**	
>70 mL/min	85 (51.2)
70–50 mL/min	58 (34.9)
<50 mL/min	23 (13.9)
**CIRS at bendamustine treatment start (%) (177)**	
<6	81 (45.8)
≥6	96 (54.2)
**Severe comorbidity § (%) (175)**	
Yes	159 (90.9)
No	16 (9.1)

* Number of analyzed patients is shown where different from total numbers of patients. § At least one CIRS item ≥4, excluding hematological comorbidities.

**Table 2:** Treatment features and supportive care. Characteristics of regimens, dose, duration, and concomitant medications for prophylaxis of treatment-related infections and cytopenias.

Characteristic (*n*. of Analyzed Patients) *	*n*. (%)
**Bendamustine containing regimen (%)**	
Bendamustine ± antiCD20	146 (81.6)
BEGEV	6 (3.4)
BAC	13 (7.3)
Other	14 (7.8)
**Bendamustine dose (%) (173)**	
90 mg/m^2^	106 (61.3)
70 mg/m^2^	58 (33.5)
<70 mg/m^2^	9 (5.2)
**Anti-CD20 monoclonal antibody (%)**	
No	17 (9.5)
Rituximab	160 (89.4)
Obinutuzumab	2 (1.1)
**N. of completed cycles (%)**	
<4	28 (15.7)
At least 4	150 (84.3)
Up to 6	120 (67.4)
**Therapy interruption (%)**	
No	142 (79.3)
Yes	37 (20.7)
**Dose delay (%) (177)**	
No	112 (63.3)
Yes (at least 1 week delay)	65 (36.7)
**Dose reduction (%) (178)**	
No	155 (87.1)
Yes	23 (12.9)
**G-CSF prophylaxis (%)**	
No	102 (57.0)
Yes	77 (43.0)
**Erythropoietin prophylaxis (%) (177)**	
No	148 (83.6)
Yes	29 (16.4)
**Trimethoprim-sulfamethoxazole prophylaxis (%) (177)**	
No	31 (17.5)
Yes	146 (82.5)
**Acyclovir prophylaxis (%) (177)**	
No	105 (59.3)
Yes	72 (40.7)

* Number of analyzed patients is shown where different from total numbers of patients.

**Table 3:** Acute and late toxicities. Details on hematologic, extra-hematologic toxicity and long term complications (secondary neoplasia) after bendamustine-based therapy. Abbreviations: UTI, urinary tract infections, CMV, cytomegalovirus, VZV, varicella zoster virus.

Adverse Event (*n*. of Analyzed Patients) *	*n*. (%)
**Hematologic**	
**Neutropenia (%)**	
No	78 (43.6)
Yes (any grade)	101 (56.4)
Grade < 3	22 (12.3)
Grade ≥ 3	79 (43.5)
**Thrombocytopenia (%)**	
No	100 (55.9)
Yes (any grade)	79 (44.1)
Grade < 3	58 (32.4)
Grade ≥ 3	21 (11.7)
**Anemia (%)**	
No	74 (41.3)
Yes (any grade)	105 (58.7)
Grade < 3	84 (46.9)
Grade ≥ 3	21 (11.7)
**Extra-hematologic**	
**Patients who had infections (%)**	
No	115 (64.2)
Yes	64 (35.7)
**Infectious events (%)**	
Respiratory infections	25 (14)
Fever of unknown origin (FUO)	13 (7.3)
Gastrointestinal infections	7 (3.9)
VZV cutaneous reactivation	6 (3.3)
UTI	5 (2.8)
Oral infections	5 (2.8)
Sepsis	5 (2.8)
CMV reactivation	2 (1.1)
Febrile neutropenia	2 (1.1)
Other	11 (6.1)
**Infectious etiology (%)**	
Bacterial	27 (15.1)
Viral	14 (7.8)
Fungal	6 (3.3)
Unknown	34 (19)
**Other toxicities (%) (177)**	
No	126 (71.2)
Autoimmune disorder (e.g., AIHA)	4 (2.3)
Skin toxicity	15 (8.5)
Other	32 (18.1)
**Admission-requiring AEs (%)**	
No	148 (82.7)
Yes	31 (17.3)
**Length of stay (days [median])**	10.00 [6.00, 20.00]
**Cause of admission (%)**	
Sepsis	9 (5)
Fever of unknown origin (FUO)	5 (2.8)
Pneumonia	4 (2.2)
Gastrointestinal complications	3 (1.7)
Cardiovascular complications	3 (1.7)
Acute kidney injury	2 (1.1)
CMV reactivation	1 (0.6)
Steven-Johnson syndrome	1 (0.6)
Other complications	3 (1.7)
**Second cancer (%)**	
No	139 (77.7)
Yes	40 (22.3)
**Second cancer type (%)**	
Hematologic cancer	10 (5.6)
Urogenital cancer	8 (4.5)
Gastrointestinal cancer	8 (4.5)
Skin cancer	7 (3.9)
Lung cancer	3 (1.7)
Breast cancer	1 (0.6)
Other	3 (1.7)

* Number of analyzed patients is shown where different from total numbers of patients.

## Supplementary Materials

The following are available online at https://www.mdpi.com/article/10.3390/jpm11040249/s1, Figure S1: univariate analysis for OS, Figure S2: univariate analysis for TTP, Table S1: Characteristics of the patients stratified according to pathological diagnosis, Table S2: Bendamustine treatment characteristics stratified by diagnosis.
