# Supplementary material for: Real Life Use of Bendamustine in Elderly Patients with Lymphoid Neoplasia
Source: J Pers Med. 2021 Mar 30;11(4):249. doi: 10.3390/jpm11040249 (PMC8066290; doi:10.3390/jpm11040249)
Supplement: Supplementary file 1 [file jpm-11-00249-s001.zip › Suppl_files_Manuscript_Dogliotti_JPM .docx]

**SUPPLEMENTARY MATERIAL**

***TABLE S1****: Characteristics of the patients stratified according to pathological diagnosis; only the most represented histologies in the enrolled cohort are shown.*

**Number of analyzed patients is shown where different from total numbers of patients.*

§*At least one CIRS item >4, excluding hematological comorbidities.*

|  | **Diagnosis** | | |  |
| --- | --- | --- | --- | --- |
| **Characteristic (n. of analyzed patients)*** | **CLL** | **FL** | **MCL** | ***p* value** |
| **n. (135)** | 51 | 43 | 41 |  |
| **Age (median [IQR])** | 74.00 [70.50, 79.00] | 74.00 [68.00, 77.00] | 73.00 [70.00, 77.00] | 0.294 |
| **Sex (%) (135)** |  |  |  |  |
| Male | 31 (60.8) | 18 (41.9) | 25 (61.0) | 0.118 |
| Female | 20 (39.2) | 25 (58.1) | 16 (39.0) |  |
| **Stage (%) (121)** |  |  |  |  |
| 0 | 12 (31.6) | 0 (0.0) | 0 (0.0) | NA |
| 1 | 11 (28.9) | 0 (0.0) | 0 (0.0) |  |
| 2 | 6 (15.8) | 2 (4.8) | 3 (7.3) |  |
| 3 | 3 (7.9) | 9 (21.4) | 1 (2.4) |  |
| 4 | 6 (15.8) | 31 (73.8) | 37 (90.2) |  |
| **Symptoms (%) (118)** |  |  |  |  |
| A | 31 (88.6) | 36 (85.7) | 31 (75.6) | 0.340 |
| B | 4 (11.4) | 6 (14.3) | 10 (24.4) |  |
| **Prognostic score type (%) (127)** |  |  |  |  |
| IPI | 0 (0.0) | 7 (17.1) | 0 (0.0) | <0.001 |
| FLIPI | 0 (0.0) | 34 (82.9) | 0 (0.0) |  |
| MIPI | 0 (0.0) | 0 (0.0) | 39 (97.5) |  |
| IPSSWM | 0 (0.0) | 0 (0.0) | 0 (0.0) |  |
| CLL-IPI | 45 (97.8) | 0 (0.0) | 0 (0.0) |  |
| other | 1 (2.2) | 0 (0.0) | 1 (2.5) |  |
| **Prognostic score risk (%) (89)** |  |  |  |  |
| Low | 0 (0.0) | 1 (2.4) | 7 (17.9) | 0.079 |
| Intermediate | 1 (11.1) | 13 (31.7) | 12 (30.8) |  |
| High | 8 (88.9) | 27 (65.9) | 20 (51.3) |  |
| **p53 disrupted (%) (29)** |  |  |  |  |
| No | 15 (57.7) | 1 (100.0) | 1 (50.0) | 1.000 |
| Yes | 11 (42.3) | 0 (0.0) | 1 (50.0) |  |
| **Total lines (median [IQR])** | 3.00 [1.00, 3.75] | 1.00 [1.00, 2.00] | 1.00 [1.00, 2.00] | <0.001 |
| **Previous ASCT (%) (134)** |  |  |  |  |
| No | 49 (98.0) | 43 (100.0) | 40 (97.6) | 0.759 |
| Yes | 1 (2.0) | 0 (0.0) | 1 (2.4) |  |
| **Bendamustine treatment line (median [IQR])** | 2.00 [1.00, 2.00] | 1.00 [1.00, 1.00] | 1.00 [1.00, 1.00] | <0.001 |
| **BCC at bendamustine treatment start (%) (124)** |  |  |  |  |
| >70 mL/min | 15 (31.9) | 28 (70.0) | 18 (48.6) | 0.006 |
| 70-50 mL/min | 24 (51.1) | 7 (17.5) | 15 (40.5) |  |
| <50 mL/min | 8 (17.0) | 5 (12.5) | 4 (10.8) |  |
| **CIRS at bendamustine treatment start (%)** |  |  |  |  |
| <6 | 17 (33.3) | 22 (53.7) | 21 (51.2) | 0.096 |
| >6 | 34 (66.7) | 19 (46.3) | 20 (48.8) |  |
| **Severe comorbidity**§ **(131)** |  |  |  |  |
| Yes | 42 (85.7) | 38 (92.7) | 39 (95.1) | 0.305 |
| No | 7 (14.3) | 3 (7.3) | 2 (4.9) |  |

***Table S2:*** ***Bendamustine treatment characteristics stratified by diagnosis.***

*Characteristics of regimens, dose, duration and concomitant medications for prophylaxis of treatment-related infections and cytopaenias according to different disease entities.*

**Number of analyzed patients is shown where different from total numbers of patients.*

| **Characteristic (n. of analyzed patients)*** | **CLL** | **FL** | **MCL** | **p** |
| --- | --- | --- | --- | --- |
| **n. (135)** | 51 | 43 | 41 |  |
| **Bendamustine containing regimen (%) (135)** |  |  |  | **<0.001** |
| Benda + antiCD20 | 46 (90.2) | 40 (93.0) | 19 (46.3) |  |
| BEGEV | 0 (0.0) | 3 (7.0) | 1 (2.4) |  |
| BAC | 0 (0.0) | 0 (0.0) | 13 (31.7) |  |
| Other | 5 (9.8) | 0 (0.0) | 8 (19.5) |  |
| **Bendamustine dose (%) (131)** |  |  |  | **0.001** |
| 90 mg/m^2^ | 25 (49.0) | 33 (82.5) | 25 (62.5) |  |
| 70 mg/m^2^ | 19 (37.3) | 7 (17.5) | 15 (37.5) |  |
| <70 mg/m^2^ | 7 (13.7) | 0 (0.0) | 0 (0.0) |  |
| **Anti-CD20 monoclonal antibody (%) (135)** |  |  |  | **<0.001** |
| No | 12 (23.5) | 1 (2.3) | 0 (0.0) |  |
| Rituximab | 38 (74.5) | 41 (95.4) | 41 (100.0) |  |
| Obinutuzumab | 1 (2.0) | 1 (2.3) | 0 (0.0) |  |
| **N. of completed cycles (%)** |  |  |  | **<0.001** |
| <4 | 16 (32.0) | 3 (7.0) | 2 (4.9) |  |
| At least 4 | 34 (68.0) | 39 (90.7) | 39 (95.1) |  |
| Up to 6 | 24 (48.0) | 36 (83.7) | 34 (82.9) |  |
| **Therapy interruption (%) (135)** |  |  |  | **<0.001** |
| No | 31 (60.8) | 38 (88.4) | 38 (92.7) |  |
| Yes | 20 (39.2) | 5 (11.6) | 3 (7.3) |  |
| **Dose delay (%) (133)** |  |  |  | 0.523 |
| No | 28 (57.1) | 28 (65.1) | 28 (68.3) |  |
| Yes (at least 1 week) | 21 (42.9) | 15 (34.9) | 13 (31.7) |  |
| **Dose reduction (%) (134)** |  |  |  | 0.092 |
| No | 42 (84.0) | 41 (95.3) | 33 (80.5) |  |
| Yes | 8 (16.0) | 2 (4.7) | 8 (19.5) |  |
| **G-CSF prophylaxis (%) (135)** |  |  |  | **<0.001** |
| No | 42 (82.4) | 17 (39.5) | 14 (34.1) |  |
| Yes | 9 (17.6) | 26 (60.5) | 27 (65.9) |  |
| **Trimethoprim-sulfamethoxazole prophylaxis (%) (133)** |  |  |  | **0.001** |
| No | 16 (32.0) | 4 (9.3) | 2 (5.0) |  |
| Yes | 34 (68.0) | 39 (90.7) | 38 (95.0) |  |
| **Acyclovir prophylaxis (%) (133)** |  |  |  | **<0.001** |
| No | 16 (32.0) | 36 (83.7) | 24 (60.0) |  |
| Yes | 34 (68.0) | 7 (16.3) | 16 (40.0) |  |
| **Erythropoietin prophylaxis (%)(133)** |  |  |  | **<0.001** |
| No | 50 (98.0) | 36 (83.7) | 25 (64.1) |  |
| Yes | 1 (2.0) | 7 (16.3) | 14 (35.9) |  |

***Table S3****:* ***Univariate analysis for OS.*** *HR, Hazard ratio; CI, confidence interval.*

**At least one CIRS item >4, excluding hematological comorbidities.*

| **Characteristic** | **HR** | **95% CI** | **p-value** |
| --- | --- | --- | --- |
| **Bendamustine dose** |  |  |  |
| 90 mg/m^2^ | — | — |  |
| 70 mg/m^2^ | 1.63 | 0.88, 3.01 | 0.12 |
| <70 mg/m^2^ | 8.61 | 3.62, 20.5 | **<0.001** |
| **Age distribution** |  |  |  |
| >80 | — | — |  |
| 65-70 | 0.11 | 0.04, 0.30 | **<0.001** |
| 71-79 | 0.40 | 0.22, 0.74 | **0.003** |
| **Diagnosis** |  |  |  |
| CLL | — | — |  |
| FL | 0.34 | 0.15, 0.79 | **0.012** |
| LPL/WM | 0.54 | 0.21, 1.42 | 0.2 |
| MCL | 0.42 | 0.19, 0.94 | **0.034** |
| MZL | 0.25 | 0.06, 1.08 | 0.063 |
| Other | 4.80 | 1.59, 14.5 | **0.006** |
| **Sex** |  |  |  |
| Male | — | — |  |
| Female | 0.54 | 0.30, 0.95 | **0.033** |
| **CIRS at bendamustine treatment start** |  |  |  |
| <6 | — | — |  |
| >6 | 2.69 | 1.43, 5.05 | **0.002** |
| **Severe comorbidity*** |  |  |  |
| Yes | — | — |  |
| No | 1.09 | 0.43, 2.74 | 0.9 |
| **ECOG** |  |  |  |
| 0 | — | — |  |
| 1-2 | 2.01 | 0.93, 4.32 | 0.074 |
| **BCC at bendamustine treatment start** |  |  |  |
| >70 mL/min | — | — |  |
| 70-50 mL/min | 4.00 | 1.86, 8.63 | **<0.001** |
| <50 mL/min | 6.36 | 2.67, 15.2 | **<0.001** |
| **Dose delay** |  |  |  |
| No | — | — |  |
| Yes (at least 1 week) | 0.92 | 0.52, 1.64 | 0.8 |
| **Dose reduction** |  |  |  |
| No | — | — |  |
| Yes | 0.76 | 0.30, 1.90 | 0.6 |
| **Therapy interruption** |  |  |  |
| No | — | — |  |
| Yes | 3.89 | 2.19, 6.89 | **<0.001** |
| **Bendamustine treatment line** |  |  |  |
| >3 | — | — |  |
| 1 | 0.25 | 0.12, 0.53 | **<0.001** |
| 2 | 0.85 | 0.39, 1.85 | 0.7 |
| **Hemoglobin at bendamustine treatment start (g/dL)** |  |  |  |
| <10 | — | — |  |
| >10 | 0.77 | 0.36, 1.65 | 0.5 |
| **Platelet count at bendamustine treatment start (10^3^/µL)** |  |  |  |
| <100 | — | — |  |
| >100 | 0.70 | 0.32, 1.49 | 0.4 |
| **G-CSF prophylaxis** |  |  |  |
| No | — | — |  |
| Yes | 0.34 | 0.18, 0.67 | **0.002** |
| **Trimethoprim-sulfamethoxazole prophylaxis** |  |  |  |
| No | — | — |  |
| Yes | 0.37 | 0.21, 0.68 | **0.001** |
| **Acyclovir prophylaxis** |  |  |  |
| No | — | — |  |
| Yes | 1.38 | 0.78, 2.43 | 0.3 |
| **Erythropoietin prophylaxis** |  |  |  |
| No | — | — |  |
| Yes | 0.72 | 0.31, 1.69 | 0.5 |

***Table S4: univariate analysis for TTP****. HR, Hazard ratio; CI, confidence interval.*

**At least one CIRS item >4, excluding hematological comorbidities.*

| **Characteristic** | **HR** | **95% CI** | **p-value** |
| --- | --- | --- | --- |
| **Bendamustine dose** |  |  |  |
| 90 mg/m^2^ | — | — |  |
| 70 mg/m^2^ | 2.49 | 1.49, 4.15 | **<0.001** |
| <70 mg/m^2^ | 5.94 | 2.29, 15.4 | **<0.001** |
| **Age distribution** |  |  |  |
| >80 | — | — |  |
| 65-70 | 0.28 | 0.14, 0.56 | **<0.001** |
| 71-79 | 0.40 | 0.22, 0.72 | **0.002** |
| **Diagnosis** |  |  |  |
| CLL | — | — |  |
| FL | 0.38 | 0.19, 0.75 | **0.006** |
| LPL/WM | 0.61 | 0.27, 1.40 | 0.2 |
| MCL | 0.39 | 0.19, 0.79 | **0.009** |
| MZL | 0.39 | 0.15, 1.02 | 0.056 |
| Other | 6.83 | 2.51, 18.6 | **<0.001** |
| **Sex** |  |  |  |
| Male | — | — |  |
| Female | 0.73 | 0.45, 1.19 | 0.2 |
| **CIRS at bendamustine treatment start** |  |  |  |
| <6 | — | — |  |
| >6 | 2.23 | 1.34, 3.72 | **0.002** |
| **Severe comorbidity*** |  |  |  |
| Yes | — | — |  |
| No | 0.86 | 0.35, 2.14 | 0.7 |
| **ECOG** |  |  |  |
| 0 | — | — |  |
| 1-2 | 0.97 | 0.45, 2.12 | >0.9 |
| **BCC at bendamustine treatment start** |  |  |  |
| >70 mL/min | — | — |  |
| 70-50 mL/min | 1.36 | 0.77, 2.40 | 0.3 |
| <50 mL/min | 2.12 | 1.05, 4.28 | **0.036** |
| **Dose delay** |  |  |  |
| No | — | — |  |
| Yes (at least 1 week) | 0.88 | 0.53, 1.45 | 0.6 |
| **Dose reduction** |  |  |  |
| No | — | — |  |
| Yes | 0.84 | 0.40, 1.77 | 0.7 |
| **Therapy interruption** |  |  |  |
| No | — | — |  |
| Yes | 3.84 | 2.25, 6.56 | **<0.001** |
| **Bendamustine treatment line** |  |  |  |
| >3 | — | — |  |
| 1 | 0.31 | 0.16, 0.60 | **<0.001** |
| 2 | 1.06 | 0.51, 2.19 | 0.9 |
| **Hemoglobin at bendamustine treatment start (g/dL)** |  |  |  |
| <10 | — | — |  |
| >10 | 0.80 | 0.42, 1.54 | 0.5 |
| **Platelet count at bendamustine treatment start (10^3^/µL)** |  |  |  |
| <100 | — | — |  |
| >100 | 1.21 | 0.58, 2.54 | 0.6 |
| **G-CSF prophylaxis** |  |  |  |
| No | — | — |  |
| Yes | 0.35 | 0.20, 0.61 | **<0.001** |
| **Trimethoprim-sulfamethoxazole prophylaxis** |  |  |  |
| No | — | — |  |
| Yes | 0.59 | 0.33, 1.08 | 0.087 |
| **Acyclovir prophylaxis** |  |  |  |
| No | — | — |  |
| Yes | 2.17 | 1.32, 3.58 | **0.002** |
| **Erythropoietin prophylaxis** |  |  |  |
| No | — | — |  |
| Yes | 0.65 | 0.31, 1.37 | 0.3 |

**Suppl. Figure 1
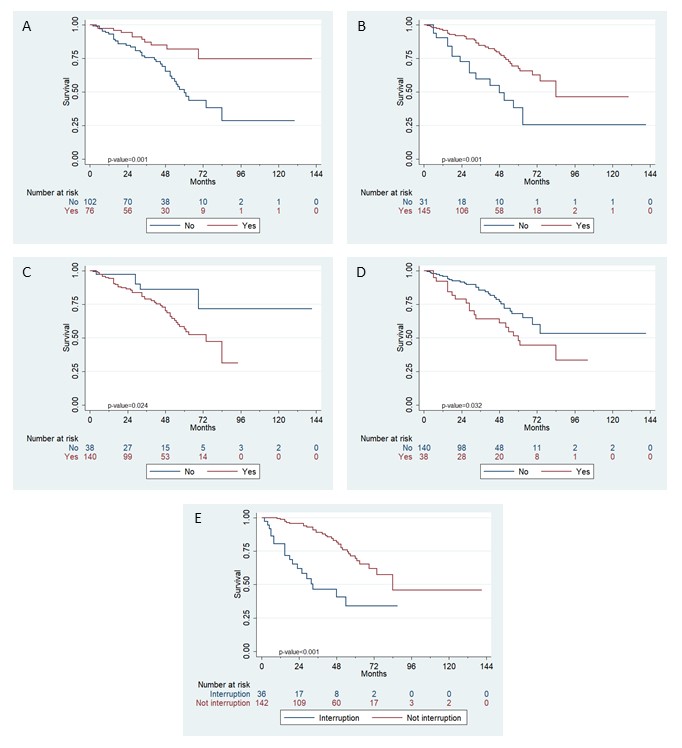
*Suppl. Figure 1: univariate analysis for OS****. panel A: median OS according to G-CSF prophylaxis use in patients receiving bendamustine; panel B: median OS according to trimethoprim sulfamethoxazole prophylaxis use in patients receiving bendamustine; panel C: median OS according to hematologic toxicity in patients receiving bendamustine; panel D: median OS according to second cancer development in patients receiving bendamustine. panel E: median OS according to bendamustine therapy interruption.*

**Suppl. Figure 2**


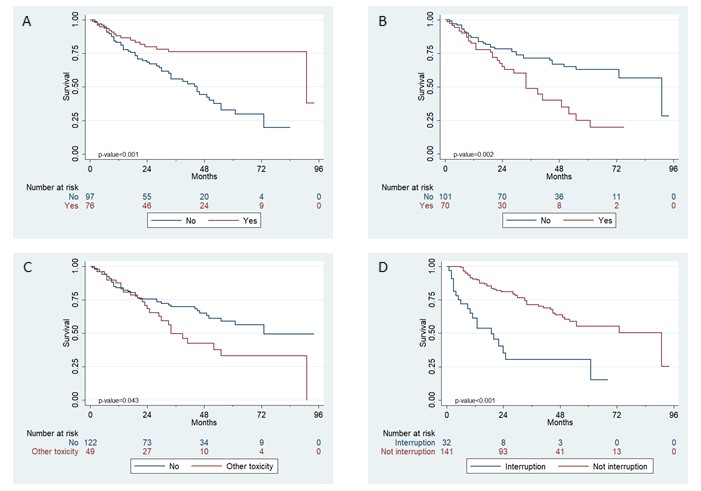


***Suppl. Figure 2: univariate analysis for TTP****. panel A: TTP according to G-CSF prophylaxis use in patients receiving bendamustine; panel B: TTP according to acyclovir prophylaxis use in patients receiving bendamustine; panel C: TTP according to non-hematologic toxicity other than infections in patients receiving bendamustine; panel D: TTP according to bendamustine therapy interruption.*
